# Increased DNA methylation levels of the insulin-like growth factor binding protein 1 gene are associated with type 2 diabetes in Swedish men

**DOI:** 10.1186/1868-7083-5-21

**Published:** 2013-11-19

**Authors:** Tianwei Gu, Harvest F Gu, Agneta Hilding, Louise K Sjöholm, Claes-Göran Östenson, Tomas J Ekström, Kerstin Brismar

**Affiliations:** 1 Rolf Luft Research Center for Diabetes and Endocrinology, Department of Molecular Medicine and Surgery, Karolinska Institute, Department of Endocrinology, Karolinska University Hospital, Stockholm SE-17176, Sweden; 2 Center for Molecular Medicine, Department of Clinical Neuroscience, Karolinska Institute, Karolinska University Hospital, Stockholm, Sweden

**Keywords:** IGFBP-1, DNA methylation, type 2 diabetes

## Abstract

**Background:**

Prospective studies have shown that low levels of circulating insulin-like growth factor binding protein-1 (IGFBP-1) are associated with the risk of type 2 diabetes. In the present study, we investigated DNA methylation in the *IGFBP1* gene to evaluate its changes in relation to serum IGFBP-1 levels in type 2 diabetes.

**Results:**

A total of 406 Swedish men, including age-matched normal glucose tolerance subjects and type 2 diabetes patients either newly diagnosed or undergoing treatment, were selected from the Stockholm Diabetes Prevention Program. *IGFBP1* methylation levels in genomic DNA extracted from peripheral blood were analysed by bisulfite pyrosequencing. Serum IGFBP-1 levels were measured by radio-immunoassay. We found that *IGFBP1* DNA methylation levels were higher in both newly diagnosed and treated type 2 diabetes patients with a mean diabetes duration of 3 years compared with subjects with normal glucose tolerance (19.8% and 20.2% vs. 16.9%, *P* < 0.001 for both). Serum levels of IGFBP-1 in newly diagnosed and in treated type 2 diabetes patients were lower compared with healthy individuals (18 μg/l both vs. 24 μg/l, *P* = 0.011, *P* < 0.001). *IGFBP1* methylation levels but not serum IGFBP-1 levels in type 2 diabetes patients were independent of body mass index. Newly diagnosed patients with a family history of diabetes (FHD) had higher *IGFBP1* methylation levels than those without FHD (20.3% vs. 18.6%, *P* = 0.017).

**Conclusions:**

This study provides the first evidence that changes in DNA methylation of the *IGFBP1* gene are associated with type 2 diabetes in Swedish men and suggests that increased *IGFBP1* DNA methylation and decreased IGFBP-1 serum levels are features of type 2 diabetes with a short duration.

## Background

Type 2 diabetes mellitus is a complex metabolic disorder influenced by genetic and environmental factors. In recent years, genome-wide association studies (GWAS) have identified a number of confirmed genetic susceptibility variants for type 2 diabetes. However, GWAS findings can only explain approximately 10% of the overall heritable risk of type 2 diabetes, which challenges our expectations for translating genetic information into clinical practice [1-3]. One of the reasons for the missing information on heritability could be that epigenetic factors are involved in the complex interplay between genes and environment. Knowledge of the epigenetic factors associated with type 2 diabetes is still limited. Therefore, epigenetic studies may provide further information to give a better understanding of the pathogenesis of type 2 diabetes [4-6].

The circulating insulin-like growth factor binding protein-1 (IGFBP-1), produced in the liver, has an inhibitory effect on the action of insulin-like growth factors and is mainly regulated by portal insulin [7,8]. Clinical investigations have demonstrated that a low circulating level of IGFBP-1 is associated with insulin resistance, type 2 diabetes and the metabolic syndrome [9-11]. We have previously analysed IGFBP-1 serum levels in Swedish middle-aged and elderly twins and found that environmental influences predominate for IGFBP-1 levels [12]. We have also demonstrated that a low serum level of IGFBP-1 predicts the development of type 2 diabetes in middle-aged Swedish men and women [9,13,14].

DNA methylation variation is hypothesized to alter individual susceptibility to type 2 diabetes [4]. DNA methylation levels are commonly analysed at clusters of CpG methylation sites in the genes and used to indicate epigenetic effects [15]. However, there are no reports from epigenetic studies of *IGFBP1* in type 2 diabetes. It is unknown whether DNA methylation patterns of the *IGFBP1* gene are associated with type 2 diabetes. In this study, we investigated DNA methylation levels of the *IGFBP1* gene in Swedish men, including subjects with normal glucose tolerance or type 2 diabetes and analysed serum IGFBP-1 levels. Our study demonstrates that increased *IGFBP1* methylation levels and reduced protein levels are associated with type 2 diabetes.

## Results

### Association of *IGFBP1* DNA methylation and IGFBP-1 serum levels with type 2 diabetes

**Figure 1:**
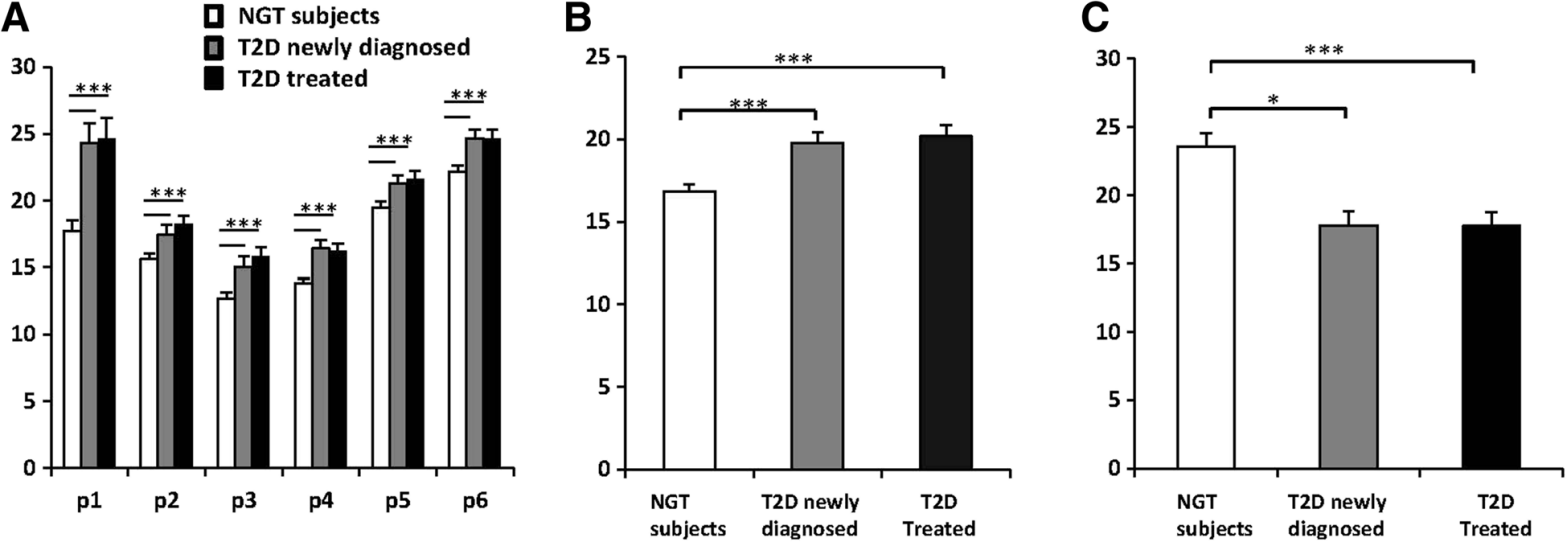
**DNA methylation and serum levels of *IGFBP1 *in Swedish men with normal glucose tolerance and type 2 diabetes. (A)***IGFBP1* DNA methylation levels at each of the six CpG sites in patients with newly diagnosed type 2 diabetes and in patients undergoing treatment were significantly higher compared with those in non-diabetic control subjects (*P* < 0.001 for all comparisons with non-diabetic subjects). **(B)** Combining all six CpG sites together as total, significantly increased DNA methylation levels of the *IGFBP1* gene in newly diagnosed and treated type 2 diabetes patients in comparison with the controls were found (18.9%, 19.8% and 16.9%, respectively, *P* < 0.001 for both type 2 diabetes group vs. controls.) **(C)** Serum IGFBP-1 levels were significantly higher in non-diabetic control subjects than in both newly diagnosed and treated patients with type 2 diabetes. *P* values: * < 0.05, *** < 0.001. IGFBP-1, insulin-like growth factor binding protein-1; NGT, normal glucose tolerance; T2D, type 2 diabetes.

We conducted genomic DNA methylation analyses of six CpG sites in the human *IGFBP1* gene. The *IGFBP1* DNA methylation levels at each of the six CpG sites were significantly higher in both newly diagnosed type 2 diabetes patients (P1 24.3%, P2 17.5%, P3 15.0%, P4 16.4%, P5 21.3% and P6 24.7%) and treated patients (P1 24.6%, P2 18.2%, P3 15.8%, P4 16.2%, P5 21.6% and P6 24.7%) compared with those in non-diabetic subjects (P1 17.7%, P2 15.6%, P3 12.7%, P4 13.%, P5 19.5% and P6 22.%) (*P* < 0.001 for all comparisons vs. non-diabetic controls) (Figure 1A). Combining all six CpG sites together, the mean values of *IGFBP1* DNA methylation levels were significantly increased in both newly diagnosed and treated patients in comparison with non-diabetic subjects (19.8%, 18.9% and 16.9%, *P* < 0.001 for both type 2 diabetes groups vs. controls) (Figure 1B). However, there were no differences in DNA methylation levels at any CpG site of the *IGFBP1* gene between newly diagnosed patients and the patients on treatments. Further analyses were performed according to the different treatments given to the treated type 2 diabetes patients. *IGFBP1* DNA methylation levels were similar in the treatment groups (20.7% in patients on physical exercise and diet control, 19.4% in patients on oral anti-diabetic drugs (OADs), 20.4% in patients on insulin treatment and 19.4% in patients on OADs + insulin).

We further analysed fasting serum IGFBP-1 levels and found that newly diagnosed and treated patients with type 2 diabetes had similar fasting serum IGFBP-1 levels (18 μg/l in both groups), which were significantly lower than those in non-diabetic control subjects (24 μg/l, *P* = 0.011 and *P* < 0.001, respectively) (Figure 1C).

### Association of *IGFBP1* DNA methylation and IGFBP-1 serum levels with family history of diabetes

**Figure 2:**
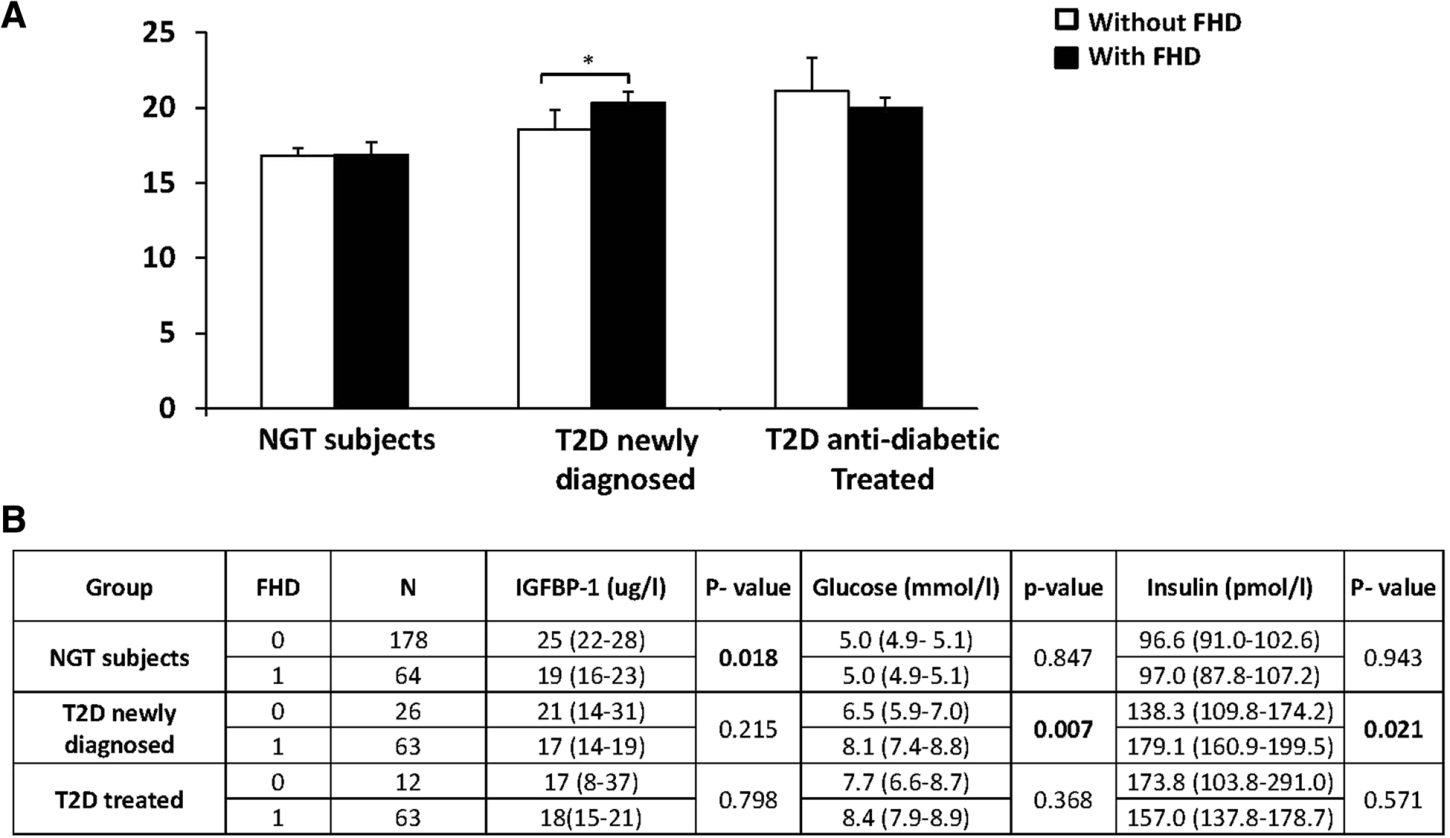
**DNA methylation and serum levels of IGFBP1 according to family history of diabetes.** Compared with those without a family history of diabetes **(**FHD) (0), non-diabetic control subjects with FHD (1) had similar *IGFBP1* DNA methylation levels (16.9% vs. 17.0%, *P* = 0.895, **A)** but significantly lower serum protein levels (19 vs. 25 μg/L, *P* = 0.018) **(B)**. Newly diagnosed type 2 diabetes patients with FHD had significantly increased *IGFBP1* DNA methylation levels (20.3% vs. 18.6%, *P* = 0.014) **(A)**, higher glucose (8.1 vs. 6.5 mmol/l, *P* = 0.007) and insulin (153.6 vs. 118.8 pmol/l, *P* = 0.022) levels compared to patients without FHD **(B)**. There were no differences in levels of *IGFBP1* DNA methylation, serum IGFBP-1, glucose or insulin between treated type 2 diabetes patients with and without FHD. *P* value: * < 0.05. FHD, family history of diabetes; NGT, normal glucose tolerance; T2D, type 2 diabetes.

To understand whether *IGFBP1* DNA methylation and serum IGFBP-1 levels are related to a family history of diabetes (FHD), we carried out comparison analyses between subjects with and without FHD. The data showed that non-diabetic control subjects with FHD had similar *IGFBP1* DNA methylation levels (16.9% and 17.0%) but lower serum protein levels compared with those without FHD (19 vs. 25 μg/L, *P* = 0.018) (Figure 2A and 2B). Newly diagnosed type 2 diabetes patients with FHD had significantly increased *IGFBP1* DNA methylation levels compared with the patients without FHD (20.3% vs. 18.6%, *P* = 0.017) (Figure 2A). Although the geometric mean values of IGFBP-1 serum levels in newly diagnosed type 2 diabetes patients with FHD appeared lower compared with the patients without FHD, no significant difference was detected. Moreover, newly diagnosed patients with FHD had higher glucose (8.1 vs. 6.5 mmol/l, *P* = 0.007) and higher insulin levels (179.1 vs. 138.3 pmol/l, *P* = 0.021) compared to patients without FHD (Figure 2B). There were no differences in the levels of *IGFBP1* DNA methylation, serum IGFBP-1, glucose or insulin between treated type 2 diabetes patients with and without FHD.

### Association of *IGFBP1* DNA methylation and IGFBP-1 serum levels with body weight

**Figure 3:**
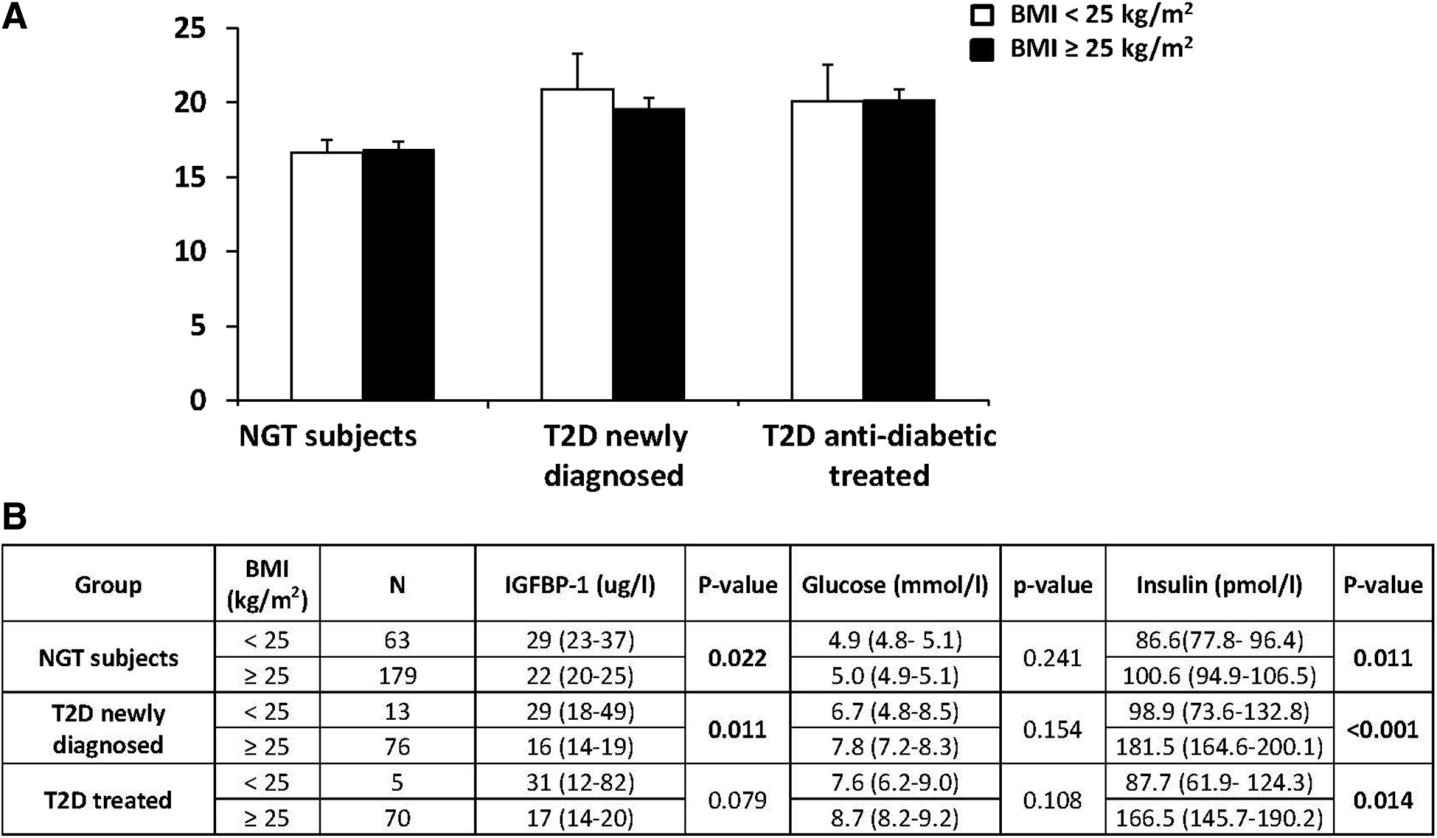
**DNA methylation and serum levels of IGFBP1 according to body mass index. (A)** No differences were found in the *IGFBP1* DNA methylation levels between lean and overweight/obese subjects in non-diabetic control subjects, newly diagnosed and treated type 2 diabetes patients. **(B)** However, lean individuals had significantly higher serum IGFBP-1, lower insulin and similar glucose levels compared with subjects who were overweight/obese. BMI, body mass index; IGFBP-1, insulin-like growth factor binding protein-1; NGT, normal glucose tolerance; T2D, type 2 diabetes.

To further investigate whether changes of *IGFBP1* DNA methylation levels were related to body weight in type 2 diabetes, we conducted analyses according to body mass index (BMI). Subjects were divided into subgroups based on a BMI cut-off of 25 kg/m^2^. There were no differences in the *IGFBP1* DNA methylation levels between lean (BMI < 25 kg/m^2^) and overweight/obese (BMI ≥ 25 kg/m^2^) subjects in any of the three groups (Figure 3A). However, compared with overweight/obese subjects, lean individuals in the control group and newly diagnosed patients had significantly higher serum IGFBP-1 levels (29 vs. 22 μg/l, *P* = 0.022; 29 vs. 16 μg/l, *P* = 0.011) and lower insulin levels (86.6 vs. 100.6 pmol/l, *P* = 0.011; 98.9 vs. 181.5 pmol/l, *P* < 0.001) (Figure 3B). No differences were found in fasting glucose levels between lean and overweight/obese subjects within any group.

We also conducted similar analyses for waist circumference with a cut-off at 94 cm. Data showed that there was no significant difference in *IGFBP1* DNA methylation levels between subjects with waists <94 cm and ≥94 cm within any group: non-diabetic control subjects (16.6% vs. 17.1%, *P* = 0.307), newly diagnosed (19.7% vs. 20.2%, *P* = 0.551) and treated type 2 diabetes patients (20.3% vs. 19.8%, *P* = 0.607).

### Correlation between *IGFBP1* DNA methylation levels and IGFBP-1 serum levels

We analysed the correlation between *IGFBP1* DNA methylation levels and IGFBP-1 serum levels. A borderline significant correlation between *IGFBP1* DNA methylation levels and IGFBP-1 serum levels was found in non-diabetic subjects (*r* = 0.186, *P* = 0.050), but not in newly diagnosed type 2 diabetes patients (*P* = 0.770) or patients undergoing treatment (*P* = 0.230).

## Discussion

We conducted an epigenetic study of the human *IGFBP1* gene in Swedish men with and without type 2 diabetes in parallel with analyses of fasting IGFBP-1 protein levels in serum. Our results demonstrated that compared with non-diabetic controls, DNA methylation levels of the *IGFBP1* gene were higher in all type 2 diabetes patients, while IGFBP-1 serum levels were lower. Newly diagnosed type 2 diabetes patients with FHD had higher DNA methylation levels compared with those without FHD. Furthermore, *IGFBP1* DNA methylation levels were found to be independent of BMI or waist circumference, while IGFBP-1 serum levels were inversely related to BMI.

It has been shown that DNA methylation is influenced by age, gender, genetic background, lifestyle and body weight [16-18]. To avoid the influence of age and gender, for our study we selected age-matched Swedish men including non-diabetic subjects, newly diagnosed type 2 diabetes patients not taking medication and patients undergoing treatment, from the Stockholm Diabetes Prevention Program (SDPP) cohort. There is a CpG island in the regions of the promoter and 5’-UTR of the *IGFBP1* gene. By using well-selected and characterized subjects, we analysed six CpG sites in 5’-UTR, which may represent the methylation levels of *IGFBP1*. A previous study has demonstrated that high CpG density region at 5’-UTR recruits a methy-CpG binding protein to the promoter and represses gene transcription [19]. We found increased DNA methylation in all type 2 diabetes patients, who also had decreased circulating levels of IGFBP-1. However, we were not able to establish the causal relationship between *IGFBP1* DNA methylation alteration and the development of type 2 diabetes from the current cross-sectional study. We further compared the *IGFBP1* DNA methylation levels among subgroups of patients on different treatments, including diet, OAD, insulin and insulin plus OAD. There were no significant differences between the subgroups, suggesting no effect of diabetes treatment on *IGFBP1* methylation levels. This could be due to the limited duration of type 2 diabetes in these patients (mean 3 years) and also that the numbers of individuals in the subgroups were relatively small.

In this study, we measured the fasting morning levels of IGFBP-1, which reflects IGFBP-1 secretion over the previous 24 hours and overall endogenous and exogenous insulin effect during the previous 24 hours [20]. Low serum IGFBP-1 levels are associated with hyperinsulinemia and subsequently with overweight or obesity [13,14]. We thus stratified the subjects according to BMI and waist circumference. The were no differences in the *IGFBP1* DNA methylation levels for any of the three groups, suggesting that the variation of *IGFBP1* DNA methylation was due to the type 2 diabetes, rather than being overweight or obese. In addition, we analysed the *IGFBP1* DNA methylation levels according to lifestyle factors including smoking status, physical activity levels and alcohol consumption. We found no significant differences within any group (data not shown).

When evaluating *IGFBP1* DNA methylation levels in type 2 diabetes patients according to FHD, we found that newly diagnosed patients with FHD not only had higher fasting glucose and insulin levels but also increased *IGFBP1* DNA methylation. This implies that there could be a trans-generation effect in patients with FHD. Furthermore, newly diagnosed patients with FHD had hyperinsulinemia and impaired glucose tolerance for a longer time before diagnosis compared with those without FHD [21].

There are limitations of the present study. First, there is the lack of human liver tissue samples for methylation analysis. We do not know whether *IGFBP1* DNA methylation levels in peripheral blood cells do reflect its methylation levels in the liver. However, we observed a weak correlation between *IGFBP1* DNA methylation levels in peripheral blood cells and circulating IGFBP-1 serum levels in non-diabetic subjects. Type 2 diabetes is a complex disease. The correlation between DNA methylation and serum protein levels is influenced by multiple factors, which may explain the lack of correlation between *IGFBP1* DNA methylation and serum IGFBP-1 levels in type 2 diabetes patients. Second, our patients had diabetes for only a short time. Therefore, the effects of diabetes treatment on DNA methylation might be too trivial to detect. Analyses of liver-tissue-specific DNA methylation levels, gene transcription in liver cell lines after RNA interference at CpG sites and using patients with longstanding diabetes are being considered for further investigation.

## Conclusions

In this study, we provide the first evidence that DNA methylation changes of the *IGFBP1* gene are associated with type 2 diabetes in Swedish men with a short duration. Taking our present study and recent reports together, we also suggest that increased *IGFBP1* DNA methylation and decreased IGFBP-1 serum levels are features of type 2 diabetes.

## Methods

### Subjects

A sample of Swedish men, comprising 242 subjects with normal glucose tolerance (non-diabetic controls) and 164 patients with type 2 diabetes, was studied. They were all participants of SDPP and lived in the municipalities of Värmdö, Upplands-Bro, Tyresö or Sigtuna in Stockholm County [22]. The subjects with normal glucose tolerance were age-matched with type 2 diabetes patients (mean age 58 years). Of the patients, 89 were newly diagnosed and not receiving medication when the clinical data were recorded. The other 75 patients had had diabetes for a mean of 3 years and were being treated with advice on physical exercise and diet control (29.3%), OADs (53.3%), insulin (5.3%) or a combination of these (4.0%). In SDPP, information on smoking status, physical activity levels and alcohol consumption were recorded for all participants based upon questionnaires [22].

All patients with type 2 diabetes in SDPP were diagnosed according to the World Health Organization (WHO) criteria [23]. FHD was defined as having at least one first-degree relative (parent or sibling) or at least two second-degree relatives (grandparents, uncles or aunts) with diabetes. The clinical characteristics of the subjects are summarized in Table 1. All data shown are means (95% CI) except fasting insulin where geometric means (95% CI) are given. *P* values from Tukey *post hoc* tests were between type 2 diabetes patients either newly diagnosed or receiving treatment and non-diabetic controls. No differences were found between newly diagnosed patients and patients receiving treatment.

**Table 1:** Clinical characteristics of Swedish men with normal glucose tolerance or type 2 diabetes

**Characteristic**	**Non-diabetic subjects**	**Patients with newly diagnosed type 2 diabetes**	**Patients with type 2 diabetes receiving treatment**
*N*	242	89	75
(FHD −/+)	(178/64)	(26/63)	(12/63)
Age (years)	58 (57–58)	58 (57–59)	59 (58–60)
BMI (kg/m^2^)	26.1 (25.9–26.3)	29.3 (28.4–30.3)***	30.5 (29.4–31.5)***
WC (cm)	93.2 (92.4–94.0)	101.8 (99.4–104.3)***	103.7 (101.2–106.2)***
Glucose (mmol/L)	5.0 (4.9–5.1)	7.6 (7.1–8.1)***	8.3 (8.1–9.0)***
Insulin (pmol/L)	96.7 (91.9–101.8)	166.1 (150.0–157.8)***	160.0 (140.0–181.8)***
SBP (mmHg)	135 (134–138)	145 (141–149)***	145 (141–149)***
DBP (mmHg)	83 (82–84)	88 (86–90)***	87 (85–89)*
IGF-I (ug/L)	171 (162–180)	162 (151–172)	156(147–166)

BMI, body mass index; WC, waist circumference; DBP, diastolic blood pressure; FHD, family history of diabetes; IGF, insulin-like growth factor; SBP, systolic blood pressure.

*P* values: * < 0.05, ** < 0.01 and *** < 0.001.

Informed consent has been received from all participants. The study has been approved by the Ethics Committee of Karolinska University Hospital and was performed in accordance with the Declaration of Helsinki II.

### DNA extraction and bisulfite treatment

We analysed human *IGFBP1* DNA methylation levels in genomic DNA samples. Genomic DNA was extracted from peripheral blood in all subjects using the Gentra Puregene Blood Kit (Qiagen, Hilden, Germany), which enables purification of high molecular weight DNA (100 kb to 200 kb) suitable for archiving. The scalable purification procedure gently removes contaminants and inhibitors and large-volume samples can be purified for genetic and epigenetic analyses. The stock solution of DNA samples was stored at −80°C until use. For epigenetic analysis, the extracted DNA samples were bisulfite treated using the EpiTect Bisulfite Kit (Qiagen). This kit gives complete conversion of unmethylated cytosine to uracil and subsequent purification in less than 6 hours. This highly sensitive method utilizes an innovative protection against DNA degradation and ensures high conversion rates of over 99%. DNA extraction and bisulfite treatment were conducted according to the manufacturer’s instructions.

### IGFBP1 DNA methylation analyses

There is a CpG island in the promoter and exon 1 of the *IGFBP1* gene. We used a PyroMark CpG assay (ENSG00000146678, Qiagen) and PyroMark PCR kit (Qiagen) for the *IGFBP1* gene methylation analysis, which included six CpG sites (**C**GAGCATCTGC**C**GC**C**G**C**GC**C**GC**C**GCCACC) at 5′-UTR of the *IGFBP1* gene as indicated with the bold letter “**C**” and referred to as P1 to P6 (P is the position). DNA methylation in mammals is normally found as 5-methyl cytosine followed by guanosine [15]. The PyroMark PCR Master Mix includes HotStarTaq DNA polymerase and the optimized PyroMark reaction buffer containing 3 mM MgCl_2_ and Deoxynucleotidse (dNTPs), 10× CoralLoad concentrate, 5× Q-Solution, 25 mM MgCl_2_, and RNase-free water. The PCR amplicon length was 177 bp and covers the sequence in human chromosome 7:45928112–45928134 (version 37.56). The methylation levels of these CpG sites were detected using the PyroMark Gold 96 Reagent Kit (Qiagen, Hilden, Germany) and the PyroMark Q96 ID pyrosequencing system (Biotage, Uppsala, Sweden). Pyrosequencing methylation analysis of CpG sites is a sensitive and accurate protocol [24,25]. PyroQ-CpG software (Biotage) was used for methylation data analysis. Unmethylated bisulfite converted and unconverted DNAs (Qiagen) were used to monitor the conversion efficiency of the bisulfite treatment and the accuracy of the methylation analyses.

### Assays for serum protein analyses

Serum samples from all subjects were included in protein analyses. Fasting serum IGFBP-1 and insulin levels were determined using in-house radio-immunoassays (RIAs) with polyclonal antibodies for human IGFBP-1 and insulin, respectively, as previously described [26,27]. The IGFBP-1 protein in serum samples stored at −20°C is stable. The intra- and inter-assay coefficients of variation (CV) values were 3% and 10%, respectively [13]. Serum IGF-1 levels were measured using an in-house RIA after acid-ethanol extraction and cryoprecipitation. To minimize interference by IGFBPs, des IGF-1 was used as a tracer as previously described [28]. The intra- and inter-assay CV values were 4% and 11%, respectively.

### Statistical analyses

Data presented in the table and figures are either means with 95% confidence interval (CI) or geometrical means with 95% CI if the data were not normally distributed. Tests for comparison of continuous variables between groups were assessed using an unpaired t-test or one-way ANOVA followed with Tukey’s *post hoc* test. Data in non-normally distributed traits were transformed to the natural logarithm to give a normal distribution before the statistical analysis was performed. Linear regression analysis was used to examine the relation between variables. A *P* value less than 0.05 was considered as significant. All data were analysed using the PASW statistic program (SPSS 20.0, Chicago, IL, USA).

## Abbreviations

BMI: Body mass index; FHD: Family history of diabetes; GWAS: Genome-wide association studies; IGF: Insulin-like growth factor; IGFBP-1: Insulin-like growth factor binding protein-1; OAD: Oral anti-diabetic drug; PCR: Polymerase chain reaction; RIA: Radio-immunoassay; SDPP: Stockholm diabetes prevention program.

## Competing interests

The authors declare that there are no conflicts of interest associated with this paper.

## Authors’ contributions

TG, HFG and KB designed the study. LKS and TJE assisted with DNA methylation. TG, HFG and AH researched and analysed the data. CGÖ collected data on subjects. TG, HFG and KB wrote the manuscript. All authors contributed to data interpretation, discussions and commented on the manuscript.
